# A quinol oxidase, encoded by *cyoABCD*, is utilized to adapt to lower O_2_ concentrations in *Rhizobium etli* CFN42

**DOI:** 10.1099/mic.0.083386-0

**Published:** 2015-01

**Authors:** Zachary R. Lunak, K. Dale Noel

**Affiliations:** Department of Biological Sciences, Marquette University, Milwaukee, WI, USA

## Abstract

Bacteria have branched aerobic respiratory chains that terminate at different terminal oxidases. These terminal oxidases have varying properties such as their affinity for oxygen, transcriptional regulation and proton pumping ability. The focus of this study was a quinol oxidase encoded by *cyoABCD*. Although this oxidase (Cyo) is widespread among bacteria, not much is known about its role in the cell, particularly in bacteria that contain both cytochrome *c* oxidases and quinol oxidases. Using *Rhizobium etli* CFN42 as a model organism, a *cyo* mutant was analysed for its ability to grow in batch cultures at high (21 % O_2_) and low (1 and 0.1 % O_2_) ambient oxygen concentrations. In comparison with other oxidase mutants, the *cyo* mutant had a significantly longer lag phase under low-oxygen conditions. Using a *cyo* :: *lacZ* transcriptional fusion, it was shown that *cyo* expression in the wild type peaks between 1 and 2.5 % O_2_. In addition, it was shown with quantitative reverse transcriptase PCR that *cyoB* is upregulated approximately fivefold in 1 % O_2_ compared with fully aerobic (21 % O_2_) conditions. Analysis of the *cyo* mutant during symbiosis with *Phaseolous vulgaris* indicated that Cyo is utilized during early development of the symbiosis. Although it is commonly thought that Cyo is utilized only at higher oxygen concentrations, the results from this study indicate that Cyo is important for adaptation to and sustained growth under low oxygen.

## Introduction

**Fig. 1.**
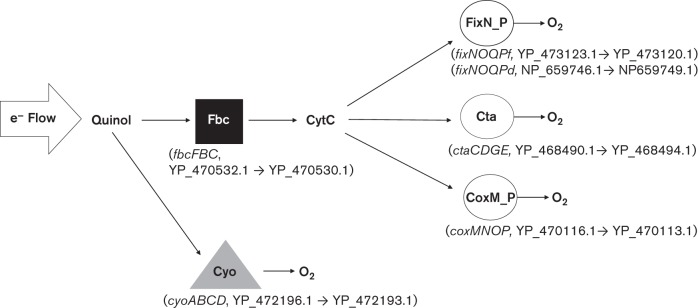
Predicted aerobic respiratory chains of *R. etli* CFN42. Electrons from quinol to oxygen can be transferred through Fbc (square), ultimately leading to cytochrome *c* oxidases (circles): FixN_P, Cta and CoxM_P. Independent of the Fbc pathway, *R. etli* can transfer electrons directly from quinol to oxygen via Cyo (triangle). In parentheses, below each of the oxidases and Fbc complex, are the indicated operons that encode each of the oxidases followed by their NCBI reference numbers of the encoded proteins. Not included in the diagram are two putative cytochrome *c* oxidases, RHE_CH00981-85 and RHE_PB00063-66.

Bacteria have remarkable adaptability to environmental changes, such as fluctuations in oxygen concentration. Presumably, an important aspect of coping with variation in oxygen concentration is that aerobic bacteria have a variety of terminal oxidases (Poole & Cook, 2000; Bueno *et al.*, 2012; Morris & Schmidt, 2013). Terminal oxidases are the enzymes that catalyse oxygen reduction during aerobic respiration. In many bacteria this step is achieved by cytochrome *c* oxidases, which catalyse electron transfer from cytochrome *c* to oxygen. Prior to this reaction, cytochrome *c* is reduced by quinol through the action of ubiquinol–cytochrome *c* oxidoreductase (Fbc), also known as the bc1 complex (Fig. 1). Quinol oxidation is a key branch point in aerobic respiration. Electrons from quinol flow either through the aforementioned Fbc or directly to oxygen via terminal oxidases known as quinol oxidases (Fig. 1). Because oxygen is a substrate for both quinol and cytochrome *c* oxidases, oxygen is expected to be a major factor in how each of these oxidases is regulated and utilized within bacteria.

The quinol oxidase encoded by *cyoABCD* (Cyo) is widespread among aerobic bacteria. Much of what is known about Cyo has been concluded from studies performed in *Escherichia coli*, where Cyo was used at high oxygen concentrations and a high-affinity quinol oxidase (Cyd), encoded by *cydAB*, was utilized at lower oxygen concentrations (D’Mello *et al.*, 1995, 1996; Tseng *et al.*, 1996). However, *E. coli* does not contain the Fbc pathway. Therefore, the regulation and utilization of Cyo may be very different in bacteria that also contain cytochrome *c* oxidases. Currently, Cyo is classified as a low-affinity oxidase along with the cytochrome *c* aa3 oxidase (Cta), encoded by *ctaCDGE* (García-Horsman *et al.*, 1994, Morris & Schmidt, 2013).

In this study, *Rhizobium etli* CFN42 was used as a model organism. Like other rhizobia, *R. etli* contains both the quinol oxidase and the Fbc pathway that contains multiple cytochrome *c* oxidases. This enables a direct comparison between quinol oxidases and cytochrome *c* oxidases within the same organism. This strain of *R. etli* only contains one quinol oxidase, Cyo, making it an ideal organism to study this particular enzyme.

Much of what is known about aerobic respiration in *R. etli* is limited to the necessary components involved in the symbiotic interaction with *Phaseolus vulgaris* (Delgado *et al.*, 1998). It has been well established that a high-affinity cbb3 cytochrome *c* oxidase (Preisig *et al.*, 1996), encoded by *fixNOQP*, is required for nitrogen fixation in *P. vulgaris*. For this reason, the regulation of this oxidase (FixN_P in Fig. 1) has been studied extensively in *R. etli*. It is tightly regulated and expressed at low oxygen (Michiels *et al.*, 1998; Girard *et al.*, 2000; Lopez *et al.*, 2001; Moris *et al.*, 2004; Granados-Baeza *et al.*, 2007). How the other oxidases in *R. etli*, such as Cyo, are utilized and regulated in *R. etli* CFN42 is unclear. Mutants, with altered levels of Cyo, have been isolated and examined in the symbiotic interaction (Soberón *et al.*, 1989, 1990). However, to our knowledge these mutations are either not in the *cyo* genes or they have not been genetically defined.

In this study, the oxygen conditions in which Cyo was utilized in liquid culture were determined. Initially, the ability of a *cyo* mutant to grow at various oxygen concentrations in comparison to other oxidase mutants was analysed. In addition, the activity of the *cyo* promoter was measured at various oxygen conditions. Lastly, the impact of *cyo* mutation on symbiosis with *P. vulgaris* was examined.

## Methods

### 

#### Bacterial strains and growth conditions.

*R. etli* strains were derived from strain CE3, a streptomycin-resistant derivative of wild-type strain CFN42 (Noel *et al.*, 1984), whose genome nucleotide sequence has been determined (González *et al.*, 2006). *R. etli* strains were grown at 30 °C on a rotating shaker in TY liquid medium [0.5 %, w/v, tryptone (Difco); 0.3 %, w/v, yeast extract (Difco); 10 mM CaCl_2_]. *E. coli* strains were grown in LB liquid medium (1.0 %, w/v, tryptone; 0.5 %, w/v, yeast extract; 0.5 %, w/v, NaCl) at 37 °C on a rotating shaker (Sambrook *et al.*, 1989). Agar medium contained 1.5 % (w/v) Bacto agar (Difco). To analyse growth at low-oxygen conditions, fully grown cultures were diluted 1 : 200 into 5 ml of TY medium resting in 60 ml serum vials. The serum vials were then capped and the headspace was flushed with nitrogen gas. Using a sterile syringe needle, ambient air (assumed 21 % O_2_) was injected back into the headspace to make it 1 and 0.1 % O_2_. For growth at 21 % O_2_, the vials were covered with aluminium foil. Cultures were then grown at 30 °C on a rotating shaker. To follow growth, 400 µl was removed from the cultures using a sterile syringe needle and the OD_600_ was analysed. In addition, for some cultures the c.f.u. ml^−1^ was enumerated. To ensure oxygen was not reintroduced into the cultures while sampling for growth, nitrogen gas was aspirated from a separate vial prior to sampling.

#### Materials and techniques for DNA isolation.

Genomic DNA was isolated from *R. etli* strains using the GenElute Bacterial Genomic DNA kit (Sigma) for use in cloning. *E. coli* NEB 5-α (Invitrogen) competent cells were transformed (Hanahan, 1983), and plasmids were isolated using QIAprep spin miniprep kit (Qiagen). DNA was recovered from agarose gels using Gel/PCR DNA Fragments Extraction kit (IBI Scientific) and modified with restriction enzymes (NEB). Custom primers were synthesized by Eurofins MWG Operon.

#### Cloning and site-directed mutagenesis.

The *fixN_P* mutant was generously provided by L. Girard (Girard *et al.*, 2000). Using PCR, *cyoA*, *cyoB*, *ctaC* and *coxN* were amplified separately from *R. etli* CE3 genomic DNA (primer sequences are listed in Table S1, available with the online Supplementary Material). PCR products were inserted into a TA cloning vector, pCR2.1. Plasmids were then digested using restriction enzymes, and DNA fragments were then inserted into plasmid pEX18Tc (Hoang *et al.*, 1998). Either the gentamicin-resistant cassette from plasmid pUCGm, the kanamycin-resistant cassette from plasmid pBSL86 or the omega chloramphenicol resistant cassette from pBSL119 was inserted (Schweizer, 1993; Alexeyev, 1995; Alexeyev *et al.*, 1995). Plasmids carrying the mutated ORFs were transferred into *R. etli* CE3 by using the plasmid-mobilizer strain MT616 on TY agar plates (Finan *et al.*, 1986; Glazebrook & Walker, 1991). CE3 transconjugants containing these constructs were selected and purified as previously described (Ojeda *et al.*, 2010). Double-crossover recombinants were screened on TY agar plates supplemented with 1 µg of tetracycline ml^−1^. Of the recombinants that were sensitive to 1 µg tetracycline ml^−1^ and resistant to 8 % (w/v) sucrose on TY agar, it was then verified by PCR that the colonies contained only the mutant allele and the wild-type allele was absent. The resulting strains are listed in Table 1.

**Table 1.** Strains and plasmids used in study

Bacterial strain or plasmid	Description, genotype or phenotype	Reference or Source
**Strains**		
*R. etli*		
CE3	Wild-type strain, *str-1*	Noel *et al.* (1984)
CE3/pZL39	CE3 carrying pZL39; Tc^R^	This study
CE119	CE3 derivative, *str-1* *fbcF* :: Tn*5*	This study
CE426	CE3 with mTn*5*SS*gusA11* at unknown site	Duelli *et al.* (2001)
CE574	CE3 derivative, *str-1 cyoA* :: Km; Km^R^	This study
CE574/pZL34	CE574 carrying pZL34; Tc^r^	This study
CE582	CE3 derivative, *str-1 coxN* :: CmΩ; Cm^R^	This study
CE583	CFNX641 derivative, *str-1 cyoB* :: Gm *fixNf* :: Sp *fixNd* :: Km; Gm^R^ Km^R^	This study
CE598	CE3 derivative, *str-1 ctaC* :: Gm; Gm^R^	This study
CE607	CE574 with mTn*5*SS*gusA11* at unknown site	This study
CFNX641	CE3 derivative; *str-1 fixNf* :: Sp *fixNd* :: Km; Sp^R^ Km^R^	Girard *et al.* (2000)
*E. coli*		
NEB-5α	Competent strain used for cloning	NEB
MT616	*pro thi endA hsdR supE44 recA-J6* pRK2013Km :: Tn*9*	Finan *et al.* (1986)
**Plasmids**		
pBSL86	*nptII* gene cassette; Km^R^	Alexeyev (1995)
pBSL119	CmΩ gene cassette; Cm^R^	Alexeyev *et al.* (1995)
pCAM111	Carries mTn*5*SS*gusA11*; Sm^R^ Sp^R^ Ap^R^	Wilson *et al.* (1995)
pCR2.1	T-A cloning vector for PCR products; Ap^R^ Km^R^	Invitrogen
pEX18Tc	Suicide plasmid; Tc^r^ *ori*T *sacB*	Hoang *et al.* (1998)
pFAJ1708	Expression vector with *nptII* promoter	Dombrecht *et al.* (2001)
pMP220	Transcriptional *lacZ* fusion vector; Tc^R^	Spaink *et al.* (1987)
pSY6	*cyoA* with Km inserted at *Sal*I site in pEX18Tc	This study
pUCGm	*aaC1* gene cassette; Gm^R^	Schweizer (1993)
pZL3	*cyoB* with Gm inserted at *Sal*I site in pEX18Tc; Gm^R^	This study
pZL12	*coxN* with CmΩ inserted at *Pst*I site; Cm^R^	This study
pZL31	Gm cassette replacing 798 bp of *ctaC* ORF; Gm^R^	This study
pZL22	*cyoB* qRT-PCR fragment cloned into pCR2.1 for standard curve	This study
pZL34	1.3 kb *Bam*HI, *Pst*I fragment with *cyoA* in pFAJ1708	This study
pZL39	pMP220 derived, 350 bp *Kpn*I, *Xba*I fragment upstream of *cyoA* fused with *lacZ*	This study

Ap^R^, ampicillin resistance; Cm^R^, chloramphenicol resistance; Gm^R^, gentamicin resistance; Km^R^, kanamycin resistance; Sm^R^, streptomycin resistance; Sp^R^, spectinomycin resistance; Tc^R^, tetracycline resistance.

#### Isolation of the *fbc* mutant.

The *fbc* mutant (CE119) was isolated via random Tn*5* mutagenesis as a *fix* mutant that still developed nodules (K. D. Noel, J. Richmond & P. Pachori, unpublished data). The genomic location of the transposon was determined to be in the *fbcF* gene of the *fbcFBC* operon (K. D. Noel & E. Rosado, unpublished data). The cytochrome *c* reduction activity was deficient in this mutant, but activity was restored after the addition of the wild-type *fbcFBC* operon (K. D. Noel & K. J. Ojeda, unpublished). In addition, an independent mutant was constructed by inserting a gentamicin cassette in the *fbcB* gene. This mutant (CE581) displayed the same phenotypic characteristics as CE119.

#### Quinol oxidase activity measurements.

Membranes were prepared and solubilized by modifying a protocol previously described (Ludwig, 1986). Cells were either grown in 500 ml Erlenmeyer flasks containing 250 ml of TY medium or 120 ml serum vials containing 10 ml of TY medium. Cells in the exponential growth phase in 250 ml of TY medium were harvested and washed with 50 mM K-phosphate buffer (pH 7.5). The washed cells were suspended in 15 ml of buffer and broken by sonication. Crude extracts were obtained by centrifugation of the sonicate for 15 min at 6 000 ***g***. The supernatant (crude extract) was removed and centrifuged at 75 000 ***g*** for 2 h 15 min. The pellet (membranes) was suspended in 3 ml of buffer. Triton-X was added to the membranes at a final concentration of 2 % and allowed to incubate on a rotator for 2 h at 4 °C to solubilize the membranes. The solubilized membranes were kept at −80 °C until tested. Measurements of quinol oxidase activity were essentially performed as previously described (Richter *et al.*, 1994). The activity of solubilized membranes was determined by measuring the change of absorbance at 275 nm. The membranes were incubated in a 1 ml reaction containing 50 mM of K-phosphate buffer (pH 7.5) and 30 µM antimycin A. The substrate, 75 µM quinol (reduced according to the method described by Rieske, 1967), was added to the mixture and the absorbance was immediately detected. The specific activity was then calculated by using the molar absorption coefficient of quinone (12,500 M^−1^ cm^−1^) and the total protein (BCA assay; Thermo Scientific) used in the reaction. To inhibit quinol oxidase activity, 1 mM potassium cyanide was added to the reaction prior to the addition of quinol.

#### *lacZ* fusion and beta-galactosidase measurements.

To generate a *cyoA* :: *lacZ* transcriptional fusion, a 350 bp fragment of the promoter region of *cyoA* was amplified from *R. etli* CE3 genomic DNA by PCR (primer sequences in Table S1). The PCR product was then inserted into the TA cloning vector, PCR 2.1. The fragment was then inserted into the pMP220 plasmid (Spaink *et al.*, 1987) at the *Kpn*I, *Xba*I restriction sites resulting in the plasmid pZL39. The plasmid pZL39 was transferred into CE3 using the MT616 plasmid-mobilizer strain. The empty vector, pMP220, was introduced into CE3 as a negative control. At different oxygen conditions (0.1–21 % O_2_), 1 ml of culture was withdrawn and washed with cold Z-buffer (Sambrook *et al.*, 1989). The beta-galactosidase assay was performed as previously described (Sambrook *et al.*, 1989).

#### Quantitative reverse transcriptase PCR (qRT-PCR).

Cultures were pelleted and immediately frozen in dry ice and stored at −80 °C. When ready for testing, cells were thawed on ice and RNA was extracted using NucleoSpin RNA II kit (Macherey–Nagel). The RNA concentration was measured by NanoDrop and 1 µg RNA was converted to cDNA using an EasyScript cDNA synthesis kit (MidSci) with the specific reverse primer for the gene of interest. As a negative control, water was added instead of the reverse-transcriptase. cDNA products were quantified by real-time PCR using EvaGreen qPCR Mastermix (MidSci), gene specific primers and the Bio-Rad iCycler. For analysing *cyo* expression, primers were designed to detect a 118 bp fragment in *cyoB* (Table S1). Samples were initially denatured at 95 °C for 10 min followed by a 40-cycle amplification protocol (95 °C for 15 s, 60 °C for 60 s). After the PCR, a melt curve was performed to ensure only one amplification product was present. The expression of the 16S rRNA gene was analysed using the same approach. Results for *cyoB* expression were normalized to the expression of the 16S rRNA gene.

#### Western blotting of BacS.

*R. etli* CE3 was grown at various oxygen concentrations (0.1–21 % O_2_) as described above. At full growth, 1 ml of cells were pelleted and resuspended in 100 µl 1× SDS buffer. The sample was then boiled for 6 min and stored at −20 °C until testing. The extracts were separated by SDS-PAGE (Laemmli, 1970) with 15 % acrylamide in the separating gel. The gel was stained with Coomassie blue (Dzandu *et al.*, 1984) to visualize protein amounts in each sample or the contents were electroblotted onto a nitrocellulose membrane. The blot was incubated with rabbit polyclonal antiserum against the BacS protein (Jahn *et al.*, 2003), and bound antibodies were detected with goat alkaline-phosphatase-conjugated anti-rabbit IgG (Sigma) that was developed with 5-bromo-4-chloro-3-indolyl phosphate.

#### Nodule assays.

Inoculation of bean seeds (*P. vulgaris*) with *R. etli* was performed as described previously (Box & Noel, 2011). For nodule staining, the wild-type and *cyo* mutants were tagged with beta-glucuronidase (GUS) by introducing pCAM111 via mating (Wilson *et al.*, 1995; Duelli *et al.*, 2001). Slicing and staining of the nodules were performed as previously described (Box & Noel, 2011). Nitrogenase activity of nodules was measured as acetylene reduction. Shoots were removed, and intact roots were incubated with acetylene in serum-capped vials. The production of ethylene was measured by gas chromatography on a Porapak N column (Noel *et al.*, 1982). The ratio of ethylene : acetylene was normalized to the collective weight of the nodules on the root to give an overall specific activity.

## Results

### Predicted aerobic respiratory branches of *R. etli* CFN42 and sequence-directed mutant construction

The genome nucleotide sequence of *R. etli* CFN42 has been determined (González *et al.*, 2006). With blast (Altschul *et al.*, 1990) searches, the terminal oxidases encoded by the genome of this strain were predicted (Figs 1 and S1). The only quinol oxidase revealed in this way was a potential Cyo quinol oxidase, encoded by contiguous *cyoABCD* genes. On the other hand, the *R. etli* CFN42 genome revealed sequences for several cytochrome *c* oxidases: FixN_P (two copies), Cta and an alternative aa3-oxidase (CoxM_P), encoded by *coxMNOP*. Two additional operons that encode putative cytochrome *c* oxidases were also revealed in the genome (RHE_CH00981-85 and RHE_PB00063-66; Fig. S1).

To gain insight into the physiological function of the oxidases, strains were constructed with mutations in the essential subunits I and/or II of the oxidases using antibiotic-resistance cassettes (Table 1). The *fixN_P* mutant, strain CFNx641, was previously constructed by Girard *et al.* (2000). In this strain both *fixNOQP* operons have been mutated. An *fbc* mutant, carrying a Tn*5* in the iron–sulfur cluster gene (*fbcF*), was also studied. Despite repeated attempts, a double mutant (*fbc*, *cyo*) was not attained. Specific mutations in RHE_CH00981-85 and RHE_PB00063-66 putative cytochrome *c* oxidases were not constructed.

### Quinol oxidase activity

**Fig. 2.**
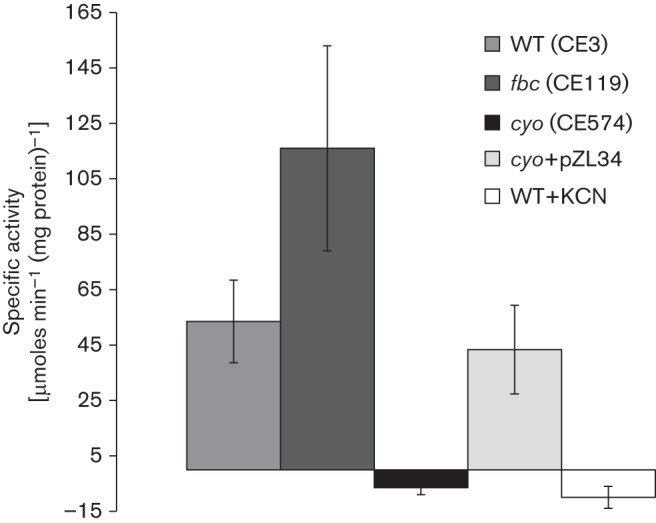
Quinol oxidase activities of solubilized membranes. Strains were grown under aerobic conditions in 250 ml of TY medium in 500 ml Erlenmeyer flasks, and solubilized membranes were prepared as described in Methods. The addition of KCN depleted activity in all strains. Reaction mixtures with no substrate (quinol) and no membranes were used as separate negative controls (data not shown) that gave no apparent quinol oxidase activity. Mean±sd values were calculated from measured activities from three separate experiments and cultures.

To confirm that the *cyo* genes encoded active proteins, and that Cyo was the only quinol oxidase present under aerobic conditions in *R. etli* CFN42, quinol oxidase activity was measured in the wild-type (CE3), *fbc* (CE119) and *cyo* (CE574) mutants during exponential growth (Fig. 2). Quinol oxidase activity was undetectable in the *cyo* mutant, supporting the notion that Cyo is the only quinol oxidase present under these conditions. Addition of the wild-type *cyo* gene (pZL34) restored activity similar to that of the wild-type. The *fbc* mutant had increased quinol oxidase activity compared with the wild-type.

### Growth at varying oxygen concentrations

To determine the growth of mutants at low oxygen, the headspace in sealed batch cultures was adjusted to 1 or 0.1 % O_2_. To ensure that low oxygen conditions had been met, BacS (Jahn *et al.*, 2003) was used as a physiological biomarker. BacS is a protein that is regulated by NifA and is expressed only at low oxygen conditions. As the oxygen concentration was lowered, the amount of BacS increased (Fig. S2).

**Fig. 3.**
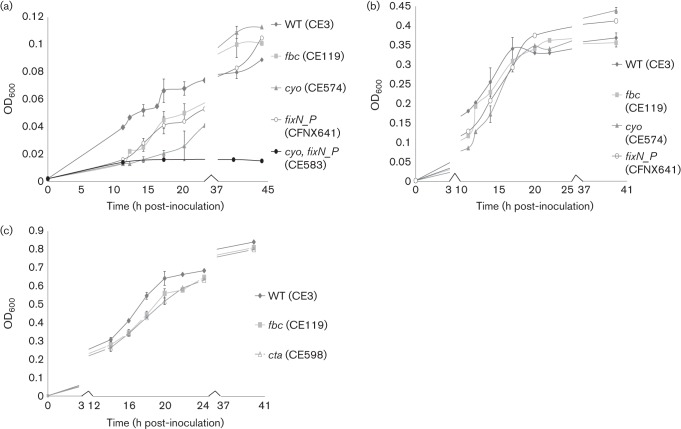
Growth curves at (a) 0.1, (b) 1.0 and (c) 21 % O_2_. Strains were initially grown in TY liquid under a gas phase with 21 % O_2_. At full growth they were subcultured 1 : 200 into 5 ml of TY medium in 60 ml serum vials. As described in Methods, nitrogen and air were added to the headspaces in the vials above the liquid to give the indicated concentrations of oxygen. Growth was followed by measuring the OD_600_. Error bars indicate sd from at least three separate experiments.

In general, growth was monitored by measuring the OD_600_ of the cultures. However, to assess whether OD_600_ was a valid indicator of growth, c.f.u. were measured in some cases as well. It was determined at low oxygen (0.1 % O_2_) that an increase in OD_600_ correlated with an increase in c.f.u. (Fig. S3). As expected, the growth of the high-affinity *fixN_P* mutant was deficient in low-oxygen conditions (Fig. 3a, b). The *fbc* mutant was defective similarly to the *fixN*_*P* mutant in low-oxygen conditions.

Unexpectedly, the *cyo* mutant was the strain that had the longest lag phase compared with the wild-type and other respiratory mutants under both 1 and 0.1 % O_2_ conditions (Fig. 3a, b). This effect was alleviated after transferring the wild-type copy of *cyoA* to the *cyo* background (Fig. S4). Furthermore, a *cyo*/*fixN*_*P* mutant (CE583) was unable to grow under the 0.1 % condition, whereas the single *cyo* or *fixN*_*P* mutant recovered to grow after a delay (Fig. 3a). The *fbc* mutant, presumably completely dependent on Cyo activity, not only suffered less delay than *cyo* but sustained its growth at 0.1 % comparably to the *cyo*, which could use FixN_P. The *cta* (CE598) and the *coxM_P* (CE582) displayed growth similar to the wild-type at low oxygen conditions (data not shown).

Under fully aerobic conditions, the *cta* mutant reproducibly had a slight growth defect (Fig. 3c). The *fbc* mutant had a similar growth defect at high-oxygen conditions. The other oxidase mutants grew similarly to the wild-type at high-oxygen conditions (data not shown).

### *cyo* expression at various oxygen concentrations

**Fig. 4.**
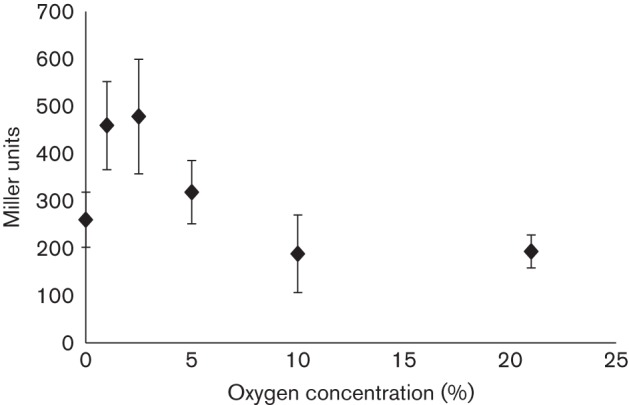
Impact of oxygen concentration on *cyo* promoter activity. Wild-type (CE3) cells, carrying the P*cyoA* :: *lacZ* transcriptional fusion (pZL39), were harvested from exponentially growing cultures at different oxygen concentrations (0.1–21 % O_2_), washed in cold Z-buffer and beta-galactosidase assay was performed. Specific activity is given in Miller units. Mean±sd values were calculated from three or more separate *lacZ* assays from two different cultures.

**Fig. 5.**
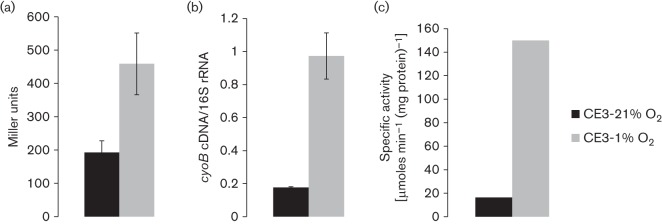
Comparison of *cyo* expression and quinol oxidase activity at low (1 %) versus high (21 %) oxygen. Wild-type cells were grown either in 21 or 1 % O_2_. Protein, RNA and membranes were all extracted from cells in the exponential phase. (a) Beta-galactosidase activity of wild-type cells carrying the transcriptional fusion plasmid, pZL39 (*cyoA* :: *lacZ*). Mean±sd values were calculated from three or more separate *lacZ* assays from two different cultures. (b) qRT-PCR of *cyoB*. RNA was extracted and converted to cDNA using the reverse gene-specific primer. As described in Methods, cDNA was then quantified by qPCR. The amount of *cyoB* cDNA (ng) was then normalized to the amount of 16S rRNA cDNA (pg) from the original RNA sample. Mean±sd values were calculated from three separate qPCR assays. (c) Specific quinol oxidase activity of wild-type solubilized membranes. Wild-type cells were grown in six separate 120 ml serum vials containing 10 ml of TY medium. These cultures were pooled together and the membranes were prepared and solubilized as described in Methods. In these experiments, growth was in serum vials rather than in Erlenmeyer flasks as described in Fig. 2.

A transcriptional fusion containing the promoter region of *cyo* and the *lacZ* reporter (P*cyo* :: *lacZ*) was used to analyse *cyo* expression at various oxygen concentrations ranging from 0.1 to 21 % O_2_ in the wild-type (Fig. 4). Expression of *cyo* gradually increased as oxygen was lowered, peaking at 1–2.5 % O_2_. The beta-galactosidase activity was approximately 2.5-fold higher from cells grown at 1 % O_2_ compared with 21 % O_2_ conditions (Fig. 5a). As the oxygen concentration was further lowered to 0.1 % O_2_, the expression decreased (Fig. 4) even though analysis of mutant growth had shown a more obvious importance of Cyo at 0.1 % O_2_ (Fig. 3a). Expression of *cyo* at 1 and 21 % O_2_ was investigated more closely using qRT-PCR, which showed that *cyo* expression was upregulated approximately fivefold at 1 % O_2_ compared with fully aerobic conditions. Depicted in Fig. 5(b) are data from one of the qRT-PCR experiments performed. Furthermore, the quinol oxidase activity in the wild-type was greatly increased at 1 % O_2_ compared with 21 % O_2_ (Fig. 5c).

### Impact of *cyo* on development of symbiosis

**Fig. 6.**
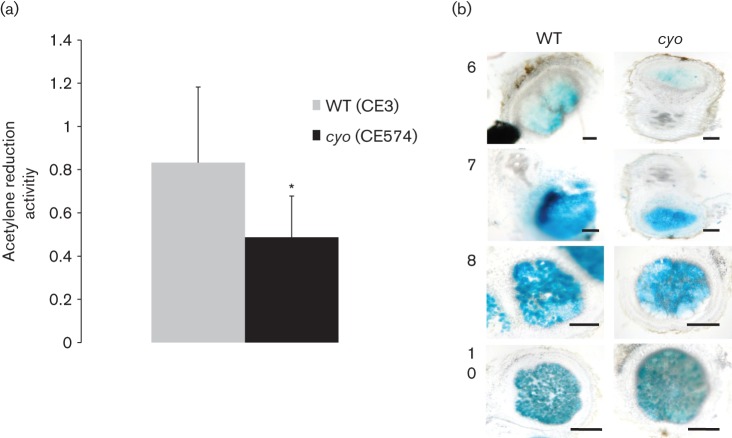
Symbiotic phenotype of *cyo*. (a) Nitrogenase activity of intact nodulated roots whose nodules harboured wild-type or *cyo* at 8 days p.i. Error bars indicate sd (*n* = 8; *P*<0.05). Acetylene reduction activity = [(ethylene peak/acetylene peak)/nodule weight]. (b) Gus-staining of nodules harbouring *gus-*tagged wild-type (CE426) and *gus*-tagged *cyo* mutants (CE607) at different days p.i. The *gusA* gene was constitutively expressed at equivalent levels (in TY culture) in both strains under low (0.1 % O_2_) and high (21 % O_2_) oxygen conditions. Therefore, staining reflects the relative density of bacterial cells. Bars in the lower right corner of each image represent 500 µm. Samples are from 6, 7, 8 and 10 days p.i.

To analyse the symbiotic role of Cyo, nodules harbouring the wild-type were compared to nodules harbouring the *cyo* mutant. Although *cyo* mutant and wild-type nodules displayed nitrogenase activity on the same day [8 days post-inoculation (p.i.)], nodules harbouring the *cyo* mutant had significantly less activity compared with the wild-type (Fig. 6a). At 9 days p.i., the nitrogenase activity was similar to the wild-type (data not shown).

As a more direct measure of infection, the bacterial content within nodules was examined by tagging the bacteria with the *gus* gene (Fig. 6b), assuming that the intensity of GUS staining was proportional to the bacterial content within the nodule. The GUS activities of the studied *gus*-tagged strains were equivalent in cells grown in TY liquid at various oxygen conditions (Fig. S5). In the early days post-inoculation (6–7 days p.i.), the staining of nodules harbouring the *gus*-tagged wild-type (CE426) was more intense compared with the nodules harbouring the *gus*-tagged *cyo* mutant (CE607). By 8 days p.i., the *cyo* nodules had comparable staining to the wild-type.

## Discussion

In this study we sought to determine the oxygen conditions in which Cyo is utilized in an Fbc-containing organism such as *R. etli* CFN42. This strain is specifically useful for studying Cyo, as inspection of the genome indicated that Cyo is the only terminal oxidase independent of the Fbc pathway. The inability to obtain a double *cyo*, *fbc* mutant under aerobic conditions supports this notion. In addition, quinol oxidase activity was undetectable in the *cyo* mutant under aerobic conditions. On the other hand, the quinol oxidase activity was increased in an *fbc* mutant, as might be predicted, given that Cyo is the only viable respiratory option in this case. These results indicate that the quinol oxidase assay is specific for Cyo in *R. etli* CFN42, and presumably enables measurement of the activity of Cyo directly under different physiological conditions. However, it is still possible that a cryptic quinol oxidase might be induced under conditions not yet studied.

To begin to understand the oxygen condition under which Cyo is utilized, we analysed the ability of the *cyo* mutant to grow at low oxygen in comparison to the wild-type and other oxidase mutants. Since the inoculant is from an aerobic culture, the results can be interpreted as the bacterium’s ability to adapt to a sudden decrease in oxygen concentration. Surprisingly, the *cyo* mutant had the greatest growth defect under low-oxygen conditions but no growth defect at high oxygen, even though Cyo is classified as a low-affinity oxidase. This result is supported by a previously reported observation in *R. etli* CFN42 that *cyo* mutants were slow to grow on minimal medium plates under micro-aerobic conditions compared with the wild-type (Landeta *et al.*, 2011). In addition, the *fbc* mutant was able to reach a similar final OD_600_ as rapidly as the *cyo* at 0.1 % O_2_. Interestingly, both *fbc* and *cyo* reproducibly reached an OD_600_ higher than that of the wild-type. The basis for this observation is unknown. These growth results, along with the upregulation of *cyo* in the wild-type, indicate that Cyo is utilized and important for adapting to and sustaining growth under low oxygen.

As predicted, the high-affinity *fixN*_P mutant had a growth defect at low oxygen and the low-affinity *cta* mutant had a growth defect at high oxygen. The *fbc* mutant had similar growth defects to the *FixN_P* mutant at low oxygen and similar growth defects to the *cta* mutant under fully aerobic conditions. This is consistent with the literature in that the Fbc pathway terminates with the aa3 oxidase under aerobic conditions and the cbb3 oxidase under micro-aerobic conditions (Bott *et al.*, 1990; Preisig *et al.*, 1996). The *coxM_P* mutant had no a distinct observable growth defect under any of the conditions tested. The two other putative cytochrome *c* oxidases (RHE_CH00981-85 and RHE_PB00063-66) were not specifically addressed in this study.

The symbiotic process from infection to bacteroid differentiation requires *R. etli* to adapt and eventually to respire at very low oxygen concentrations. Based on the results from growth in liquid cultures, it was hypothesized that Cyo may have a role during infection, because it is required for the bacterium to grow while adapting to lower levels of oxygen. The onset of nitrogen fixation was initially tested, as it was assumed that the more efficiently the bacteria infected, the faster they would differentiate into bacteroids and fix nitrogen. Although wild-type nodules and *cyo* nodules started to fix nitrogen at the same time, the wild-type nodules showed significantly more nitrogenase activity compared with the *cyo* nodules at the earliest time investigated. A similar result was reported in *Bradyrhizobium japonicum*, where a mutant defective for a Cyo homologue (*coxWXYZ*) displayed an approximately 30 % decrease in nitrogenase activity (Surpin and Maier, 1999). However, in the present study there was no significant difference in nitrogenase activity after 8 days p.i. Using *gus*-tagged bacteria, the results show that there was significantly less bacterial content in early-developed *cyo* nodules (6–7 days p.i.). As nodules matured, the staining was similar between the wild-type and *cyo* nodules. Taken together, these results indicate that Cyo is advantageous during the early stages of symbiosis but has little role in the later stages. A logical inference is that Fbc-dependent oxidases are not sufficient to provide optimal bacterial growth during the infection phase.

A potential reason for observing such gross phenotypes associated with Cyo is the fact that *R. etli* CFN42 does not contain the high-affinity Cyd quinol oxidase. However, studies in other rhizobial species have revealed that Cyo may be utilized at lower oxygen conditions regardless of the presence of Cyd. Two separate studies in *Sinorhizobium meliloti,* using DNA microarray and transcriptional fusion respectively, indicated that *cyo* is transcriptionally upregulated (2–4-fold) at lower oxygen concentrations (1–2 % O_2_) (Trzebiatowski *et al.*, 2001; Bobik *et al.*, 2006). In *B. japonicum*, based on cyanide inhibitor titration patterns of cell membranes, the Cyo homologue (*coxWXYZ*) is predicted to be expressed under micro-aerobic conditions (1 % O_2_) (Surpin *et al.*, 1996). In addition, a mutation in *coxWXYZ* had a growth defect at low oxygen levels under chemolithotrophic conditions (Surpin & Maier, 1998). To our knowledge these organisms contain a Cyd quinol oxidase based on blast searches. It has yet to be ruled out whether the absence of Cyd has had an impact on the manner in which Cyo is utilized and expressed in *R. etli* CFN42. Nevertheless, our results indicate that Cyo and Cta oxidases have distinct physiological roles in regard to oxygen. Based on our growth studies, Cyo enhances the ability to respire at lower oxygen concentrations in comparison with the Cta oxidase. Furthermore, it appears that Cyo is capable of functioning in batch culture at 0.1 % O_2_ as effectively as the FixN_P oxidase.

We propose that Cyo is a versatile oxidase that can function under a broad range of oxygen concentrations based on the growth results of the *fbc* mutant, in which it is assumed that Cyo is the only functional oxidase. With only slight deficiency in both instances, the *fbc* mutant was able to grow under both fully aerobic and low oxygen conditions. On the other hand, Cta and FixN_P seem more specialized with respect to the oxygen concentrations at which they support growth. The *cyo*, *fixN_P* mutant was unable to grow at 0.1 % O_2_, indicating that Cta is unable to function at this oxygen concentration. Under fully aerobic conditions, a *cyo*, *cta* double mutant was unattainable indicating that other cytochrome *c* oxidases are in sufficient for growth under fully aerobic conditions. A recent study on the alphaproteobacterium *Gluconobacter oxydans* also indicates that Cyo is capable of functioning under a wide range of oxygen conditions as a *cyo* mutant had a defect in growth and oxygen consumption under both free oxygen and oxygen-limiting conditions (Richhardt *et al.*, 2013). This bacterium is an obligate aerobe yet it contains neither cytochrome *c* oxidases nor a high-affinity Cyd quinol oxidase, indicating that it may be taking advantage of the versatility of Cyo that we have shown in the present study.

Although it is capable of functioning under a wide range of oxygen concentrations, Cyo may be most important at intermediate oxygen levels. Based on our results, *cyo* expression peaks at approximately 1–2.5 % O_2_. Perhaps at these concentrations, oxygen is too low for Cta to be adequately effective but not low enough to induce FixN_P. Having an oxidase such as Cyo that is capable of functioning at various oxygen concentrations would be of great benefit for many bacteria, particularly soil bacteria that frequently have to adjust to wide ranges of oxygen conditions.
